# Sustained Effects of Multi-Modal Educational Interventions on Pediatric Emergency Department Utilization Patterns

**DOI:** 10.51894/001c.164042

**Published:** 2026-07-31

**Authors:** Destiny Kanning

**Affiliations:** 1 MSU College of Nursing

## 24

### Background

Pediatric emergency department (ED) overcrowding is increasingly driven by non-emergent visits, contributing to system strain, prolonged wait times, and resource inefficiencies. Caregiver uncertainty regarding symptom severity and limited health literacy may influence care-seeking behaviors. This study evaluated the impact of a multi-modal educational intervention on caregiver health literacy and pediatric ED utilization patterns.

### Methods

A community-engaged educational initiative was implemented across multiple Michigan hospital regions, including structured seminars, multilingual print materials, and digital resources focused on symptom recognition and care navigation. Caregiver health literacy and confidence were assessed using pre- and post-intervention surveys. Pediatric ED visit data were analyzed longitudinally before and after implementation to evaluate changes in non-emergent utilization. Statistical analyses included paired comparisons for survey responses and trend analysis of utilization patterns across defined pre- and post-intervention periods.

### Results

A total of 523 caregivers participated in survey assessments. Post-intervention, caregiver-reported confidence in determining appropriate care settings significantly improved compared to baseline (*p* < 0.01). Health literacy scores increased by 12 percentage points following educational exposure. Longitudinal ED data demonstrated an 18.6% reduction in non-emergent pediatric visits across participating regions during the post-implementation period compared to baseline trends. Temporal alignment between intervention rollout and utilization decline suggests an association between education exposure and care-seeking behavior changes.

### Conclusions

Multi-modal, community-engaged educational interventions were associated with improved caregiver health literacy and measurable reductions in non-emergent pediatric ED utilization. These findings support patient-centered education as a scalable systems-level strategy to mitigate ED overcrowding while strengthening informed care navigation.

